# Sentieon DNASeq Variant Calling Workflow Demonstrates Strong Computational Performance and Accuracy

**DOI:** 10.3389/fgene.2019.00736

**Published:** 2019-08-20

**Authors:** Katherine I. Kendig, Saurabh Baheti, Matthew A. Bockol, Travis M. Drucker, Steven N. Hart, Jacob R. Heldenbrand, Mikel Hernaez, Matthew E. Hudson, Michael T. Kalmbach, Eric W. Klee, Nathan R. Mattson, Christian A. Ross, Morgan Taschuk, Eric D. Wieben, Mathieu Wiepert, Derek E. Wildman, Liudmila S. Mainzer

**Affiliations:** ^1^National Center for Supercomputing Applications, University of Illinois at Urbana-Champaign, Urbana, IL, United States; ^2^Department of Research Services, Mayo Clinic, Rochester, MN, United States; ^3^Department of Executive IT Administration, Mayo Clinic, Rochester, MN, United States; ^4^Department of Health Sciences Research, Mayo Clinic, Rochester, MN, United States; ^5^Carl R. Woese Institute for Genomic Biology, University of Illinois at Urbana-Champaign, Urbana, IL, United States; ^6^Department of Crop Sciences, University of Illinois at Urbana-Champaign, Urbana, IL, United States; ^7^Genome Sequence Informatics, Ontario Institute for Cancer Research, Toronto ON, Canada; ^8^Department of Biochemistry and Molecular Biology, Mayo Clinic, Rochester, MN, United States; ^9^Department of Molecular and Integrative Physiology, Mayo Clinic, Rochester, MN, United States

**Keywords:** Sentieon, variant calling, benchmarking, GATK, DNASeq

## Abstract

As reliable, efficient genome sequencing becomes ubiquitous, the need for similarly reliable and efficient variant calling becomes increasingly important. The Genome Analysis Toolkit (GATK), maintained by the Broad Institute, is currently the widely accepted standard for variant calling software. However, alternative solutions may provide faster variant calling without sacrificing accuracy. One such alternative is Sentieon DNASeq, a toolkit analogous to GATK but built on a highly optimized backend. We conducted an independent evaluation of the DNASeq single-sample variant calling pipeline in comparison to that of GATK. Our results support the near-identical accuracy of the two software packages, showcase optimal scalability and great speed from Sentieon, and describe computational performance considerations for the deployment of DNASeq.

## Introduction

Advancements in sequencing technology (Metzker, 2010; Goodwin et al., 2016) have resulted in an explosion of Whole Genome Sequencing (WGS) and Whole Exome Sequencing (WES) (Stephens et al., 2015). As sequencing machines become faster and cheaper (Illumina, 2018b), analysis must speed up as well. It is becoming less and less acceptable for genomic variant calling to take many hours, let alone days, on a single WGS sample. This is important for both medical applications where rapid genomic analysis could mean accurate diagnosis in time to save a patient’s life, as well as in agriculture where massive genotyping efforts are used to refine breeding programs *via* genomic prediction. Yet the standard software package for variant calling, GATK (Genome Analysis Toolkit; Van der Auwera et al., 2013), still requires many hours to analyze a single WGS sample, even on multiple nodes and after optimization (Heldenbrand et al., 2018).

Since 2014, Sentieon’s DNASeq pipeline (Sentieon, 2018) has been promoted as an ultrafast alternative to GATK (Weber et al., 2016; Freed et al., 2017). Other ultrafast options, such as Genalice (Plüss et al., 2017) and Isaac (Raczy et al., 2013), do not adhere to the original and trusted algorithms of GATK. Additionally, Genalice replaces the commonly used file formats BAM and VCF with the proprietary GAR, which takes hours to convert back to BAM when needed. However, Sentieon claims to follow the GATK Best Practices (DePristo et al., 2011; Van der Auwera et al., 2013) through reimplementation of GATK’s algorithms in C, C++, Python, and ASSEMBLY. It thus constitutes a highly optimized rewrite of the Java-based GATK, and also includes an optimized version of the popular BWA-MEM aligner (Li, 2013) prompting us to choose it for evaluation. Unlike the open-source GATK, however, DNASeq requires a license for use.

Here we present the results of unbiased benchmarking of DNASeq by an independent academic group. Our investigations focused on the broadly applicable and clinically useful case of single-sample variant calling. Speed, accuracy, scalability, and resource utilization were measured. Evaluations of DNASeq’s speed and accuracy are presented in comparison to GATK3.8 and GATK4 (single-threaded), the two most recent versions of GATK at the time of writing. The results suggest that Sentieon offers superior compute speed to GATK without loss of accuracy.

## Materials and Methods

### Experimental Setup

#### Software Versions

All DNASeq tests were run using Sentieon version 201711.02, except for one BWA MEM performance benchmark run on version 201711.03. A trial license was provided by Sentieon. GATK3.8 was downloaded from the Broad Institute’s software download page (Broad Institute, 2018), build GATK-3.8-0-ge9d806836. Picard version 2.17.4 and GATK 4.0.1.2 were downloaded from GitHub as pre-compiled jar files. For alignment comparisons, we used BWA-MEM v0.7.16, samtools view from Samtools v1.5, and Novosort from Novocraft v3.04.06.

#### Tools Benchmarked

We benchmarked “best practices” single-sample germline variant calling pipelines built with GATK3.8, GATK4.0, and Sentieon DNASeq (**Table 1**; see *Tool Comparison Overview: Sentieon vs GATK* for exceptions). Both GATK and DNASeq haplotyping steps offer a ploidy option, making them equally applicable to non-human sequencing data. Alignment was performed by BWA-MEM or its optimized alternative from Sentieon (marked with †). A sample benchmarking script can be found at https://github.com/ncsa/Sentieon_DNASeq_Benchmarking.

**Table 1:** Sentieon DNASeq vs. GATK pipelines.

Pipeline Step	Sentieon	GATK 3.8/4.0
Alignment	BWA MEM^†^	BWA MEM
Sorting	Sort utility	NovoSort
Deduplication	LocusCollector and Dedup	MarkDuplicates
Realignment	Realigner	*Not recommended*
Quality score recalibration	QualCal	BaseRecalibrator
Apply new quality scores	*Invoked during Haplotyper*	PrintReads (3.8)/ApplyBQSR (4.0)
Variant callling	Haplotyper	HaplotypeCaller

Sentieon’s optimized BWA MEM marked with †.

#### Data

Three datasets were used in testing: 1) A dataset corresponding to whole genome sequencing (WGS) of NA12878 (Zook et al., 2014; Zook et al., 2018) to ∼20X depth was downloaded from Illumina BaseSpace on Dec 16, 2016. The NA12878 Illumina Platinum variant calls were used as the truth set to assess variant calling accuracy on these reads. 2) A dataset corresponding to WGS of NA24694 (Church, 2005) was downloaded on January 19, 2018 from Genome in a Bottle (JIMB, 2018). The NA24694 data arrived in multiple files, which we combined into several subsets to mimic sequencing depths of 25X, 50X, 75X, and 100X, as recommended in the ftp data download README for the NA24694 dataset. 3) A small synthetic dataset simulating WGS on chromosomes 20-22 was created using NEAT-genReads (Stephens et al., 2016; Stephens, 2018), a synthetic reads generator. The software introduced random mutations into the hg38 reference, and the simulated reads were produced from that mutated reference. The mutations were recorded in a “Golden VCF,” which was used as the truth set when assessing variant calling accuracy on these synthetic data. The command used to generate synthetic reads and the Golden VCF can be found on the github repository mentioned in the Data Availability section. The October 2017 GATK bundle was used for the human reference (hg38), dbSNP (build 138), and the Mills and 1000G gold standard indels.

#### Hardware

All tests were conducted on Skylake Xeon Gold 6148 processors with 40 cores, 2.40 GHz. Each node had 192 GB and 2,666 MHz RAM. The nodes were stateless, connected to a network-attached IBM GPFS ver. 4.2.1 with custom metadata acceleration. The cluster used EDR InfiniBand with 100 Gb/sec bandwidth, 100 ns latency. Nodes ran Red Hat Enterprise Linux 6.9. Each test was run on a single node. We ran two to three replicates of most tests; the differences across the replicates were negligible, and we are confident that the walltime was not affected by other activity on the cluster.

### Tool Comparison Overview: Sentieon vs GATK

Sentieon DNASeq tools largely mirror those of GATK (**Table 1**), and in both pipelines the steps can be individually skipped or replaced by other tools. Although GATK no longer recommends realignment, we included Sentieon’s Realigner tool in all runs except for those intended to compare directly to GATK because realigning can convey benefits in a Sentieon pipeline. While GATK creates a separate recalibrated BAM by default using PrintReads (GATK3.8) or ApplyBQSR (GATK4.0), Sentieon’s default is to apply BQSR calculations “on the fly” during the Haplotyper step. Although users do have the option to generate a recalibrated BAM with DNASeq’s ReadWriter algorithm, this step is extraneous to Sentieon’s recalibration process. Therefore, there is no equivalent to GATK’s PrintReads/ApplyBQSR tool in our DNASeq pipelines.

## Results

### Thread-Level Scalability

**Figure 1:**
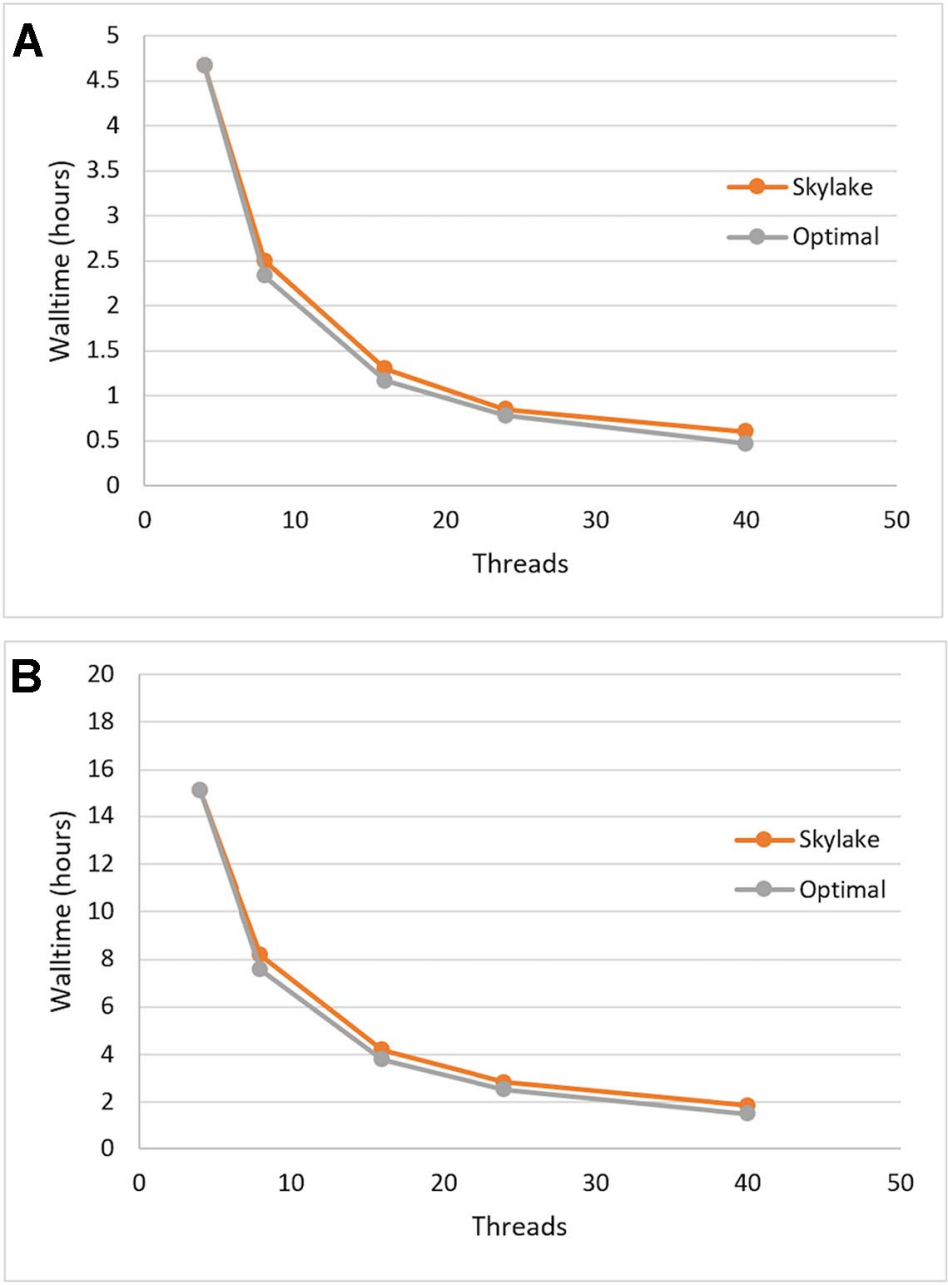
Sentieon DNASeq pipeline: demonstrated scaling across threads on Skylake architecture vs. optimal (linear) scaling. Sample: NA12878, WGS, 20X. Data points reflect averages over two replicates, highlighting **(A)** post-alignment steps only and **(B)** the full pipeline including alignment.

We tested the single-node scalability of Sentieon DNASeq by running the same pipeline with 4, 8, 16, 24, and 40 (max) threads. Optimal scalability was calculated by projecting the walltime decrease proportionately to the increase in thread count, using the first walltime measurement at 4 threads as the starting point. Each of the constituent tools appeared to scale equally well (data not shown), and the entire DNASeq pipeline scaled near-optimally up to our max of 40 threads/node (**Figure 1**). The pipeline completed in ∼16 h when running across 4 threads, and ∼2 h when running across 40.

### Effect of Sequencing Depth on Performance

**Figure 2:**
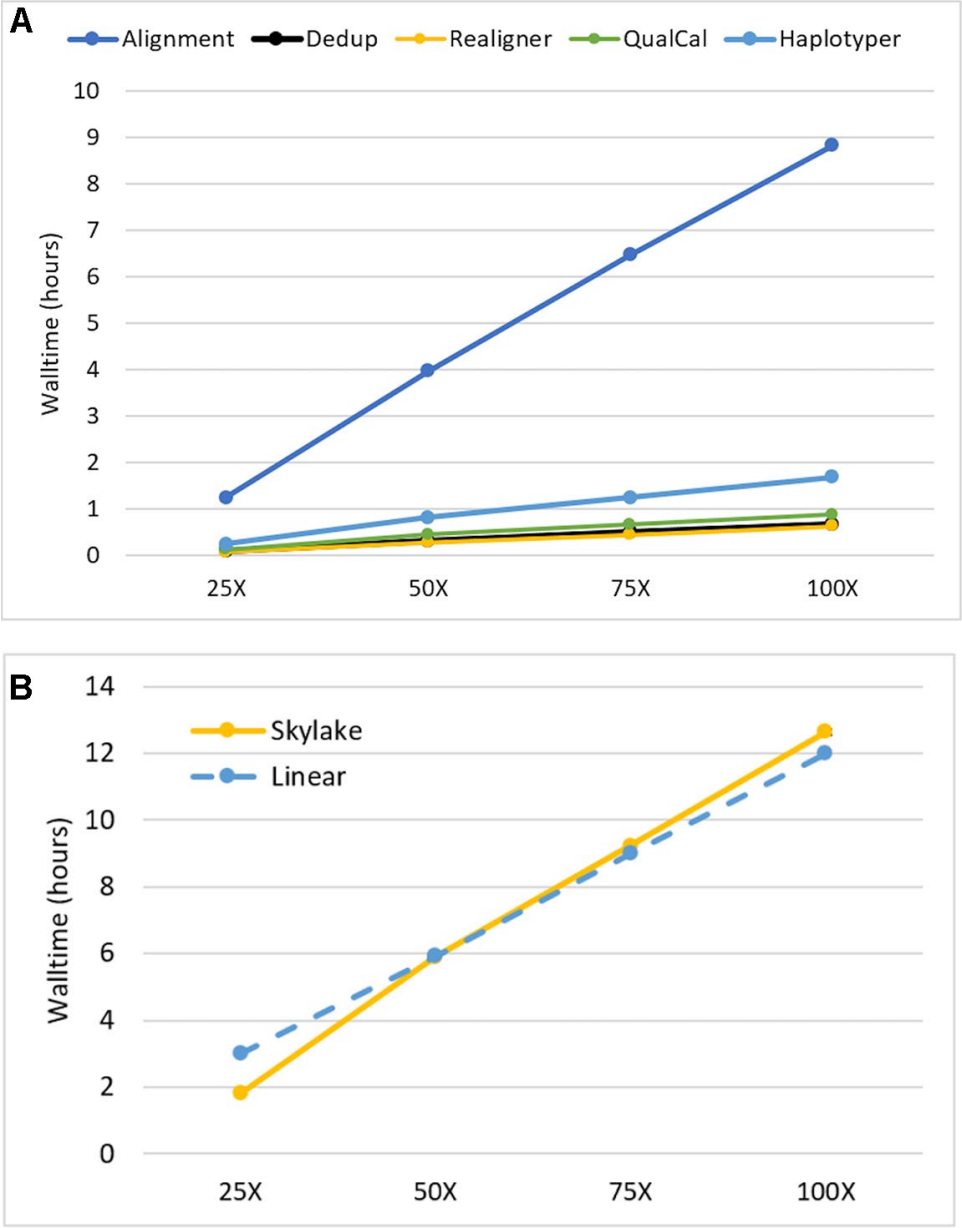
Sentieon DNASeq scalability as a function of sequencing coverage depth **(A)** by tool and **(B)** across the entire pipeline. Sample: NA24694, WGS, 25X-100X. Datapoints reflect averages over two replicates. Error bars are included in **(B)** but are too small to be visible.

We investigated the impact of sequencing depth on performance by running our Sentieon DNASeq pipeline on NA24694 WGS data subsets representing 25X, 50X, 75X, and 100X coverage depth. We ran each subset on the maximum available cores/node (40) to minimize runtime. All individual tools in the pipeline, including alignment, appeared to scale equally well. (**Figure 2**, top). The pipeline as a whole showed a small drop-off in performance between 25X and 50X: walltime increased more than twofold on 50X depth, compared to the 25X dataset, which is half the size (**Figure 2**, bottom). However, it scaled near-linearly at all depths beyond 25X. Linear scaling was calculated using the median walltime measurement by projecting that an n-fold increase or decrease in depth would lead to an equivalent n-fold increase or decrease in runtime. We do not believe this scaling pattern is due to any specific feature of alignment, as it also holds for the post-alignment portion by itself (data not shown). Considering the I/O-intensive nature of the DNASeq workflow (described below), it is more likely that the 25X data were not large enough to impact performance on our filesystem, resulting in lower run time. Nonetheless, the overall walltime performance remains outstanding: on data sequenced at 100X depth, the pipeline completed in fewer than 13 h. We expect these results to apply, qualitatively if not numerically, to other human data sequenced using Illumina technology. In other species, such as polyploid plants, which have greater content of highly repetitive genomic regions, the scalability pattern might be different due to the extra work required for read realignment, and higher content of multiply mapped reads. An in-depth investigation of these differences was deemed out of scope for this manuscript.

### Computational Performance Relative to GATK

To understand the extent of the performance improvements introduced by Sentieon, we compared the runtime of GATK vs. DNASeq on NA12878 WGS data. The computational performance of GATK3.8 and GATK4.0 have been reviewed in detail by Heldenbrand et al. (2018). We ran each of the three pipelines with their respective default settings and maximum thread count (40) to establish a “baseline.” Then we ran each pipeline with “optimal” settings: for DNASeq, 40 threads across the pipeline (same as baseline), and for GATK3.8 and GATK4, the optimized parameters recommended in Heldenbrand et al. (2018) (**Table 2**, reproduced with permission). No data-level parallelization was applied, and each test was performed on a single node. This choice was driven by the fact that both GATK 3.8 and DNASeq do not support cross-node parallelization *via* MPI, and the GATK4 Spark-enabled cross-node parallelization was not yet mature enough for testing at the time we ran our experiments.

**Table 2:** Summary of optimized parameter values for GATK3.8 and GATK4.0 in reference to parallel garbage collection (PGC) threads, tool threads, async I/O, and AVX threads.

Tool name	GATK3.8		GATK4.0		
	PGC	Tools threads	PGC	Async	AVX threads
MarkDuplicates	2 threads	1	2 threads	N/A	N/A
BaseRecalibrator	20 threads	−nct 40	20 threads	Yes for Samtools, No for Tribble	N/A
ApplyBQSR	Off	−nct 3	Off		N/A
HaplotypeCaller	Off	−nt 1 – nct 39	Off		8

Reproduced from Heldenbrand et al. (2018) with permission.

Our walltime comparison excludes BWA MEM (not part of GATK; see *Comparison of Sentieon BWA to Traditional BWA*) and realignment (as the GATK team recommends against it). In this configuration, DNASeq completed in less than half an hour on NA12878 WGS 20X, while the GATK pipelines took between 15 and 25 h on the same dataset (**Table 3**).

**Table 3:** Speed comparison: Sentieon DNASeq vs. GATK.

Pipeline	Walltime (h)	Sentieon Speedup
DNASeq	.49	–
GATK3.8 Baseline	21.7	x44
GATK3.8 Optimized	15.3	x31
GATK4.0 Baseline	24.9	x51
GATK4.0 Optimized	20.7	x42

Speedup factor indicates n-fold speedup represented by DNASeq walltime as compared to GATK walltime. Sample: NA12878, WGS, 20X.

### Comparison of Sentieon BWA to Traditional BWA

Sentieon DNASeq includes an optimized version of BWA MEM and a proprietary utility for sorting an aligned SAM into a BAM. We juxtaposed Sentieon’s BWA MEM and sort utility with a typical alignment and sorting pipeline consisting of traditional BWA MEM (Li, 2013), samtools view (Li et al., 2009), and NovoSort (Novocraft Technologies, 2014). We compared performance of the two by running both with 40 threads on NA12878 WGS data (no piping). The Sentieon version was ∼28% faster: 1.25 h vs. 1.73 h. This speed-up results from optimization of the klib library, at the cost of almost doubling the memory used by BWA: 22.45 ± 1.58 GB vs 12.13 ± 0.56 GB, measured as the resident set size (no swapping to disk was observed).

#### Version 201711.03

A new version of Sentieon (201711.03) was released as we completed our testing, featuring a non-trivial speed-up of BWA MEM derived from a complete rewrite of the traditional BWA MEM code. This new version completed in 0.95 h, 25% faster than 201711.02 and 45% faster than “BWA MEM → samtools view → NovoSort.”

### Variant Calling Accuracy

The accuracy of Sentieon’s DNASeq pipeline has been demonstrated in several FDA and DREAM Challenges (U.S. Food and Drug Administration, 2016a; U.S. Food and Drug Administration, 2016b; Sage Bionetworks, 2016). As the algorithms underlying Sentieon are nearly identical to those underlying GATK, the two can be expected to produce similar results. Nevertheless, we ran a cursory comparison of Sentieon’s DNASeq accuracy against GATK4. NA12878 and the synthetic chr 20-22 dataset were run through both pipelines. The resultant VCFs were compared to their respective truth sets and to each other, using the vcf-compare tool from the NEAT package (Stephens, 2018). The comparison was limited to the Illumina Platinum confident regions (Illumina, 2018a). In comparing Sentieon and GATK4 directly, we treated the output from GATK4 as the truth set because DNASeq is based on GATK algorithms. The command used to run comparisons is available on github.

The output of vcf-compare provides counts for true positives (*TP*), false positives (*FP*), and false negatives (*FN*). To account for both precision (*p*) and recall (*r*), we calculated F-scores based on these values using the formula $F_{1}=\frac{2pr}{p+r}$, where $p=\frac{\mathrm{TP}}{\mathrm{TP}+\mathrm{FP}}$ and $r=\frac{\mathrm{TP}}{\mathrm{TP}+\mathrm{FN}}$. Precision and recall values were similar for each comparison.

Sentieon and GATK4 were highly concordant with each other (**Table 4**) on both datasets, as expected. Using realignment in DNASeq did not meaningfully affect concordance with GATK4; the difference could become more significant on datasets of poorer quality. Both toolkits had high rates of variant detection relative to the truth sets. Significantly, GATK and Sentieon demonstrated effectively identical detection rates on the Illumina Platinum data.

**Table 4:** Variant detection accuracy in Sentieon DNASeq and GATK4: *F*_1_ scores.

Dataset	Synthetic WGS, chr 20-22	NA12878
Sentieon vs. GATK4	0.99	0.997 w/ Realigner
		0.997 w/o Realigner
**Truth sets**	**Golden VCF**	**Illumina Platinum variant calls**
Sentieon vs. Truth set	0.96	0.96
GATK4 vs. Truth set	0.95	0.96

### Computer Resource Utilization

Variant calling workflows are notorious for high RAM utilization, high rates of disk I/O, and inefficient data access patterns, all of which can lead to performance issues (Banerjee et al., 2016; Kathiresan et al., 2017). Therefore, we investigated the patterns of compute resource utilization for DNASeq in addition to its speed and accuracy. We recorded CPU load, memory utilization and I/O patterns for each tool while running the DNASeq pipeline on the NA12878 dataset. Our in-house profiling utility memprof (Kindratenko, 2018) accesses/proc/PID for each process it monitors.

#### CPU Utilization

**Figure 3:**
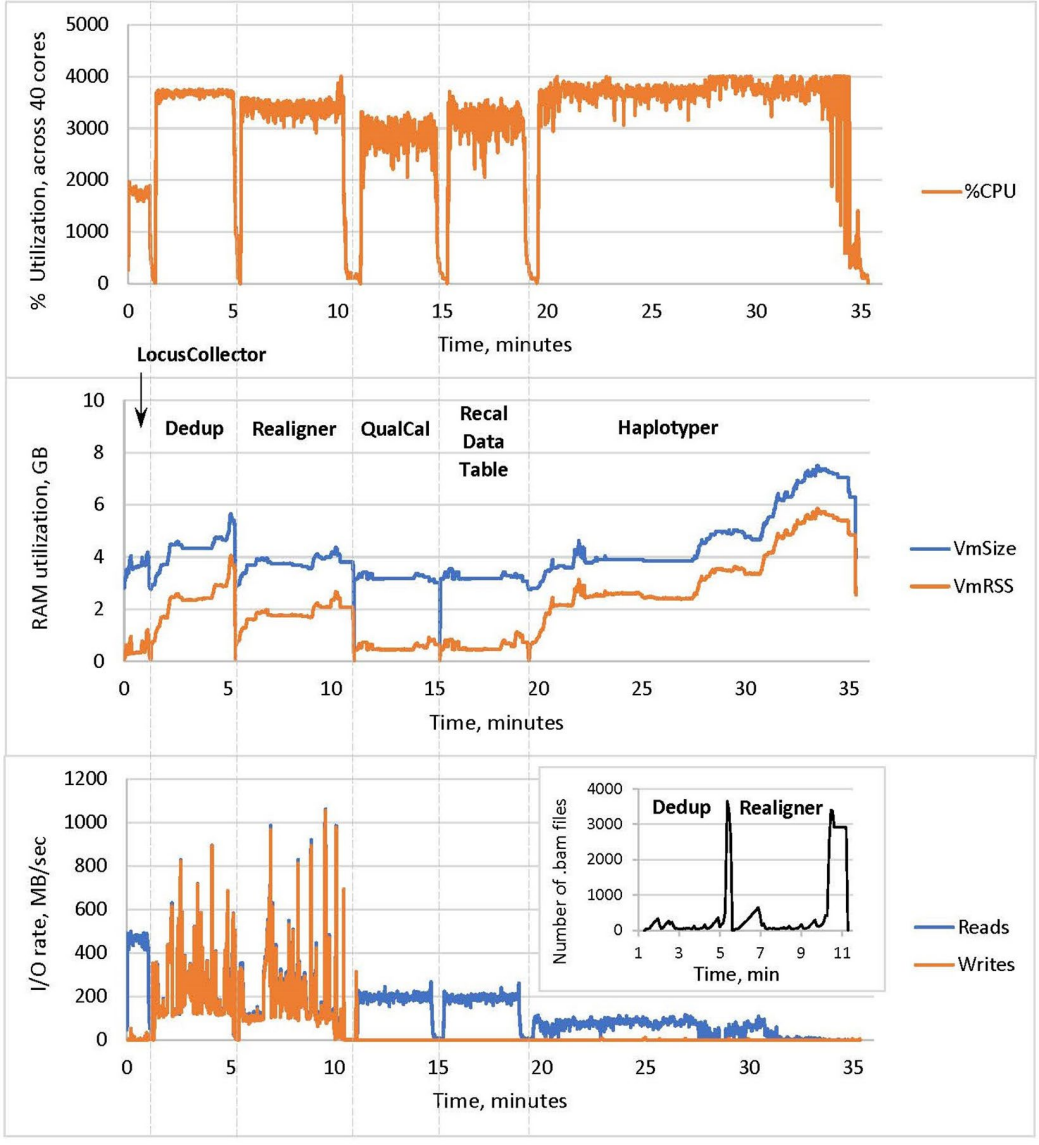
CPU utilization, memory usage and I/O of the Sentieon DNASeq tools, excluding BWA MEM. The pipeline steps are labeled in the middle panel, following the –algo options used in the script. CPU utilization in the top panel corresponds to the sum total across the 40 cores on the node. RAM utilization in the middle panel was measured as resident set size (VmRSS) and total RAM reserved for computation (VmSize). I/O rates in the bottom panel were measured in reads and writes per second. Sample: NA12878, WGS, 20X.

The resulting profile of DNASeq (**Figure 3**) shows near-maximum core utilization by all tools except LocusCollector (one of the algorithms used for deduplication). This suggests that the tools are largely CPU-bound, explaining the strong thread-level scalability described above.

#### RAM Utilization

Haplotyper uses the most memory in the pipeline (up to 6 GB), as expected for the local reassembly subroutine. High RAM utilization toward the end of the process is likely due to processing of the difficult HG38 decoy regions. Nonetheless, DNASeq RAM utilization across all tools is lower than some previous GATK benchmarks (Chapman, 2014) have shown.

#### I/O Rates

Deduplication and realignment show very active and similar I/O patterns, reaching high rates for both reads from the input BAM and writes to the output BAM. Many intermediary BAM files are created during these steps, resulting in high data-level parallelization at the cost of high I/O (**Figure 3**, inset in the bottom panel). This could introduce a filesystem bottleneck when analyzing large numbers of samples (hundreds) simultaneously on a cluster, but can be countered by using local disk or SSDs instead of network storage. In contrast, no new BAMs are created during QualCal (BQSR), as it only calculates the required modification of the quality scores and the actual recalibration is applied during the variant calling stage. Thus the rate of writes/sec is very low for QualCal. We also include the near-identical profile for the optional command that applies the recalibration to calculate the post calibration data table (**Figure 3**, step **Recal Data Table**). For comparison, GATK’s I/O performance was extensively measured and reviewed by Banerjee et al. (2016).

## Discussion

The tests we conducted were intended to a) benchmark Sentieon’s DNASeq speed and scalability and b) compare DNASeq to GATK as an alternative option for germline variant calling. Our results show that DNASeq is fast: for a WGS sample sequenced to approximately 20X depth, the DNASeq pipeline processes from FASTQ to VCF in under 2 h, and from aligned sorted BAM to VCF in less than half an hour. This opens up possibilities for point-of-care patient analysis in the clinic, massive reanalysis of legacy data and high throughput variant calling in livestock and crop breeding programs.

Our scaling tests show that DNASeq scales optimally across threads, which suggests that it will run efficiently on a variety of processors. Our tests were limited to single-node, multi-threaded scaling, although both single-threaded and multi-node tests may be of future interest. DNASeq also scales well as sequencing depth increases, a useful characteristic as deeper sequencing becomes more common. There does appear to be a small slowdown between 25X and 50X, however, which suggests that DNASeq may be most efficient on less deeply sequenced samples.

When compared to GATK, we found Sentieon DNASeq to be equally accurate. Sentieon uses the same algorithms as GATK and reliably puts out new releases in response to GATK version updates. Comparisons to Illumina platinum calls for NA12878 yielded equivalent results, suggesting no meaningful differences in reliability. It is possible that more discrepancies may be revealed when measuring with different metrics.

In terms of runtime, GATK post-alignment processing can take up to a day. Even with parameter optimizations in place, we found GATK3.8 took 15 h, while GATK4 (single-threaded) took 20 h. DNASeq is able to complete the same work over 30x faster, representing a time savings of approximately 97%. This increase in speed doesn’t appear to rely on increased resource consumption: While Sentieon’s BWA MEM implementation did utilize twice as much RAM as traditional BWA MEM, we found that RAM utilization among the other DNASeq tools did not exceed previous results recorded for GATK. Overall, our results suggest that Sentieon’s DNASeq pipeline does present a viable alternative to GATK, offering significantly better speed than GATK3.8 and GATK4 without sacrificing accuracy.

## Author’s Note

The authors of this paper did not receive any benefits or compensation, financial or otherwise, from Sentieon in exchange for testing DNASeq, evaluating its performance, or expressing positive views thereof.

## Data Availability

The NA12878 and NA24694 datasets analyzed in this study can be found in the appropriate Genome in a Bottle repositories:

NA12878: ftp://ftp-trace.ncbi.nlm.nih.gov/giab/ftp/data/NA12878/NIST_NA12878_HG001_HiSeq_300x
NA24694: ftp://ftp-trace.ncbi.nlm.nih.gov/giab/ftp/data/ChineseTrio/

The synthetic dataset representing WGS on chromosomes 20–22 will be made available by the authors, without undue reservation, to any qualified researcher.

Benchmarking scripts and commands used to generate synthetic data and accuracy comparisons are available at https://github.com/ncsa/Sentieon_DNASeq_Benchmarking.

## Author Contributions

LM designed the tests, drawing on previous work with MT. KK conducted the investigation; JH contributed additional research. KK and LM wrote the manuscript and prepared the visuals. The project was administered by DW and LM. Results were discussed with SB, MB, TD, SH, MEH, MH, MK, EK, NM, CR, EW, and MW.

## Funding

Major funding was provided by the Mayo Clinic Center for Individualized Medicine and the Todd and Karen Wanek Program for Hypoplastic Left Heart Syndrome.

## Conflict of Interest Statement

The authors declare that the research was conducted in the absence of any commercial or financial relationships that could be construed as a potential conflict of interest.
